# Advanced Technology in a Real-World Rehabilitation Setting: Longitudinal Observational Study on Clinician Adoption and Implementation

**DOI:** 10.2196/60374

**Published:** 2024-12-30

**Authors:** Louise Michelle Nettleton Pearce, Julie Pryor, Jason Redhead, Catherine Sherrington, Leanne Hassett

**Affiliations:** 1 Royal Rehab Group Sydney Australia; 2 Institute for Musculoskeletal Health Sydney Local Health District Sydney Australia; 3 Sydney School of Health Sciences Faculty of Medicine and Health The University of Sydney Sydney Australia; 4 Susan Wakil School of Nursing and Midwifery The University of Sydney Sydney Australia; 5 Sydney School of Public Health Faculty of Medicine and Health The University of Sydney Sydney Australia; 6 Implementation Science Academy Sydney Health Partners Sydney Australia

**Keywords:** rehabilitation, technology, digital health, virtual reality, robotics, exoskeleton device, implementation science, physiotherapy, physical therapy, occupational therapy, mobile phone

## Abstract

**Background:**

Advanced technologies are becoming increasingly accessible in rehabilitation. Current research suggests technology can increase therapy dosage, provide multisensory feedback, and reduce manual handling for clinicians. While more high-quality evidence regarding the effectiveness of rehabilitation technologies is needed, understanding of how to effectively integrate technology into clinical practice is also limited. Current implementation of rehabilitation technology is inconsistent, with low uptake among clinicians and frequent reports of technology abandonment. An Australian rehabilitation provider opened a new technology therapy center in 2022, offering a unique opportunity to generate practice-based evidence to inform future technology implementation and research.

**Objective:**

This study aimed to investigate the implementation and adoption of advanced technology within a real-world rehabilitation setting.

**Methods:**

This study was a longitudinal observational study in a rehabilitation organization with inpatient, outpatient, and community settings. Allied health clinicians (n=119) within the organization had access to advanced technologies, with patients receiving neurological, spinal cord injury, brain injury, or general rehabilitation. Interventions included 21 advanced technologies, including robotic, virtual reality (VR), sensor-based, and functional electrical stimulation devices. Clinicians received training for devices in a staged approach by external and internal trainers. Data were collected from patient electronic medical records from July 1, 2022, to June 30, 2023. Outcomes included frequency of advanced technology use, patient demographics (age, gender, and primary health condition), clinician discipline, rehabilitation service (inpatient, outpatient, or community), goals of technology therapy sessions, and therapy dosage achieved (minutes active, number of repetitions, and meters walked).

**Results:**

Clinicians used advanced technology 4208 times with 269 patients over 12 months; specifically, physiotherapists (2716/4208, 65%), occupational therapists (1396/4208, 33%), and allied health assistants (96/4208, 2%). The majority of patients had stroke, spinal cord injury, or brain injury diagnoses (188/269, 70%). Devices were typically used to target impairment and activity limitation–related goals. Frequently used devices included gait training body-weight support (VR treadmill and overground), overground robotic exoskeletons, and upper limb robotic VR devices. Outpatient services were the dominant users of advanced technology (3940/4208, 94%). Clinicians most commonly used devices for patients with stroke (1973/4208, 47%) and the greatest variety of devices for patients with stroke and spinal cord injury. The relative use of lower limb robotic devices was greater in inpatient services (91/178, 51%, vs outpatient services, 963/2335, 41%) (χ^2^_1_=6.6, *P*=.01) and for patients with spinal cord injury (48/95, 51%, vs all other conditions, between 24%-31%; χ^2^_5_=16.8, *P*=.005).

**Conclusions:**

The type and amount of advanced technology use differed between patient populations and rehabilitation settings. To support clinician use of advanced technology, devices should match the rehabilitation context. Tailored strategies are important, such as clinician training. Further practice-based research is required to provide guidance on implementation and to establish the effectiveness of advanced technology use.

## Introduction

There is burgeoning interest in harnessing the power of digital interventions to enhance health care services [1]. Such interventions have potential to help address the growing need for rehabilitation worldwide [2,3]. Within rehabilitation, this may involve devices such as virtual reality, robotics, smartphone apps and activity trackers [4]. While evidence is emerging, research has shown digital interventions can improve patient outcomes (eg, in mobility and upper limb function) [5-9], improve patient engagement in rehabilitation [10-13], and increase therapy dosage [6,14,15].

As technologies advance and become more affordable [16-18], they are increasingly accessible in rehabilitation [10,19,20]. Despite this, low device uptake among clinicians and frequent reports of technology abandonment persist [19,21-24]. Addressing these issues is imperative to avoid wasting health care resources on unsuccessful technology implementation efforts [24,25]. With a paucity of research conducted within rehabilitation settings, key issues include a poor understanding in selecting devices for clinical settings, who to use devices with and how to effectively integrate technology with current rehabilitation practices [1,19,26-28].

A new technology therapy center was opened by an Australian-based rehabilitation provider in 2022. This study is based within the center, which incorporates a range of advanced technologies, defined by the provider as “new technological interventions used in therapy to empower physical, cognitive, social or communicative skill development.” Advanced features include robotic componentry to maneuver limbs (eg, powered and nonpowered exoskeleton and end-effector devices) [29,30], immersive and nonimmersive virtual reality (VR) [6,31], multichannel functional electrical stimulation (FES), built-in sensors which provide real-time feedback, or a combination of these features.

Current evidence for advanced technologies is in its infancy, demonstrating potential benefits for additional therapy dosage [6,8,15,32], multisensory feedback and reducing physical demands on therapists, especially when working with patients who have little to no active motor control in major muscle groups [4,13,22,30]. In the landscape of rapidly evolving technology, research cannot afford to wait for effectiveness to be definitively established before examining implementation within clinical settings. For complex interventions, evidence of effectiveness alone is not adequate to facilitate integration into practice due to the interplay of factors such as device novelty, substantial learning requirements, and extensive organizational adaptation [24,33]. Investigating the real-world implementation of a range of advanced technologies presents a unique opportunity to advance this research field, generating practice-based evidence to directly inform technology use within clinical rehabilitation and future large-scale trials [34].

The aim of this study was to investigate the implementation and adoption of advanced technology within a real-world clinical rehabilitation setting. Specific research questions regarding this rehabilitation setting were as follows:

1. Which advanced technologies did clinicians use?
2. Who used the advanced technology?
3. Why did clinicians use advanced technology?
4. What therapy dosage did patients achieve with advanced technology?

## Methods

### Study Design

This longitudinal observational study of usual care is reported according to the Reporting of Studies Conducted using Observational Routinely-collected health Data (RECORD) statement [35].

### Setting and Participants

The study site is a combined private-public rehabilitation setting in Australia which serves adults with neurological and nonneurological conditions, specializing in spinal cord and brain injury. Rehabilitation services include 5 combined outpatient and community services, 3 inpatient units, and 2 community only services. The technology therapy center opened in July 2022 and all allied health clinicians in the organization (n=119) had access to devices.

Clinicians received training on devices before and after center opening. Training requirements for each device varied and are outlined in Multimedia Appendix 1; training timeframes ranged from 2 hours to more than 35 hours. Various models of training were used, including external trainers, web-based modules, internal train-the-trainer, and communities of practice. Clinicians had clinical support, ongoing training, and technical support through an internal advanced technology clinical lead and ad hoc support from technology suppliers.

### Intervention: Advanced Technologies

Between September and October 2020, the advanced technology clinical lead, senior allied health clinicians and management collaborated to finalize a list of technologies for purchase, based on current Australian Therapeutic Goods Administration–approved rehabilitation devices and using a selection criteria tool [36]. Another element of device selection was data security. Devices were only selected if data could be stored locally or on secure cloud-based software in Australia. Devices were acquired between November 2020 and February 2022. At center opening, 25 advanced technologies were available, 21 of which were physical rehabilitation devices. Multimedia Appendix 1 contains details of these devices, categorized in Textbox 1.

**Device classifications categorized by lower limb, upper limb and other devices.**

**Lower limb devices (n=8)**

- Body-weight support treadmill with virtual reality (BWS-T-VR)
- Body-weight support overground (BWS-OG)
- Robotic overground (Robotic-OG)
- Sensor-based virtual reality (Sensors-VR)
- Robotic treadmill body-weight support virtual reality (Robotic-T-BWS-VR)
- Robotic functional electrical stimulation (robotic functional electrical stimulation [FES])

**Upper limb devices (n=9)**

- Robotic-virtual reality (VR)
- Sensors-VR

**Other devices (n=4)**

- Augmented VR
- Immersive VR
- Advanced FES

### Data Sources and Variables

Data were recorded by clinicians and extracted weekly through a Business Intelligence report, which searched all patient electronic medical records (Kyra Clinical, Telstra Health, Melbourne, Australia) organization-wide from July 1, 2022, to June 30, 2023. Progress note entries which included an “advanced technology therapy session” template were eligible for inclusion. This template includes device-specific subheadings to prompt clinicians to record device data (refer to Multimedia Appendix 2) and was introduced as usual practice before commencing data collection. Our only exclusion criterion was any progress note entry which detailed use of a device not outlined in Multimedia Appendix 1 (eg, single or dual-channel electrical stimulation devices), resulting in exclusion of 54 progress note entries. Data extracted included: session date, patient details (medical record number, age, gender, primary health condition, and clinical service), clinician discipline, device used, location, technology session goals, duration (total time, active time [eg, walk time]) and steps, repetitions, or meters walked. In addition to medical record data, nonidentifiable service data regarding number of staff trained per device were collected. Variables used for analysis are detailed in Textbox 2.

**Details of variables used for analysis.**

**Types of advanced technologies**

- Advanced technologies used categorized into lower limb, upper limb, or other, with subcategories outlined in Textbox 1

**Patient data**

- Age, gender, and primary health condition

**Clinician and rehabilitation service**

- Clinician discipline and rehabilitation service type categorized into inpatient (onsite), outpatient (onsite; including combined outpatient and community services, or community services who saw patients onsite), or community (offsite)

**Goals of advanced technology therapy session**

- Clinician-reported therapy session goals coded and categorized according to the International Classification of Functional, Disability and Health (ICF) framework [37], as addressing “impairment” (in body function or structure), “activity limitation,” or “participation restriction”

**Therapy dosage achieved**

- Therapy dosage was calculated based on active therapy time (minutes spent active during the session), number of repetitions completed (number of repetitions completed of an exercise or number of steps, where one step is equivalent to one repetition [38]), or number of meters walked (if applicable)

### Statistical Analysis

Data analysis for this study was conducted in R (version 4.3.1; R Core Team) and consisted of summary statistics to describe device use by device category, patient characteristics, clinician discipline, and clinical service, each month and across the 12-month period. Missing data within included progress note entries were also recorded and summarized descriptively. Chi-square tests of independence were conducted to investigate associations between type of device used and patient primary health condition, as well as rehabilitation service. A significance level of .05 was used for chi-square tests.

### Ethical Considerations

Ethics approval for this study was provided by Northern Sydney Local Health District Human Research Ethics Committee on May 19, 2022 (2022/ETH00364). A waiver of consent was granted to collect data from patients’ electronic medical records, including patients’ medical record numbers to allow analysis of the number of sessions and variety of different technologies used by each patient. However, no personal information regarding patients or clinicians was collected and no individual participants are identifiable in any findings presented in this study.

## Results

### Overall Advanced Technology Use and Staff Training

Across the 12 months, clinicians used advanced technologies 4208 times with 269 patients (14% of all patients). All rehabilitation services within the organization accessed devices for patient therapy sessions. In total, 80 allied health clinicians (80/119, 67%) were trained across devices (n=41 outpatient and community clinicians, n=29 inpatient clinicians and n=10 clinicians working across inpatient, outpatient, and community services); including 35 physiotherapists, 33 occupational therapists, and 4 allied health assistants.

### Which Advanced Technologies Did Clinicians Use?

**Figure 1:**
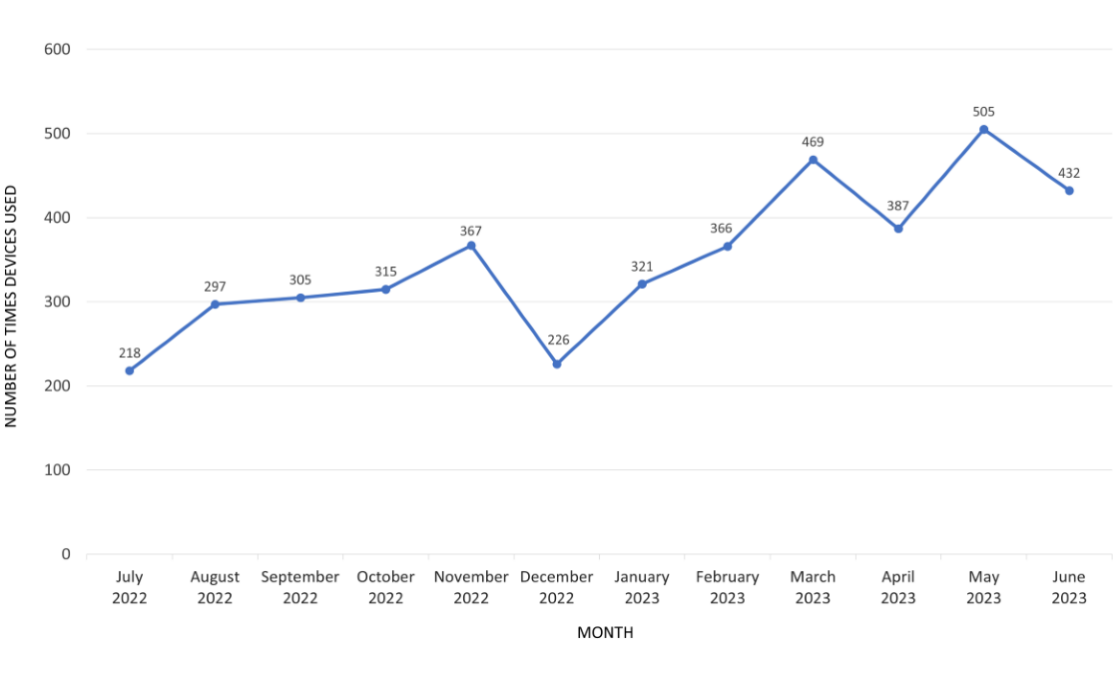
Monthly device use across the 12-month period (July 2022 to June 2023).

Our data captured clinicians using 20 of 21 (95%) physical rehabilitation devices. Monthly device use gradually increased, excluding holiday months (Figure 1).

Lower limb devices were most frequently used (2513/4208, 60%). The most used device was an augmented VR treadmill with optional BWS (580/4208, 14%). Devices for upper limb training were also frequently used (1270/4208, 30%); most commonly, a robotic VR device for distal upper limb joints (374/4208, 9%). Table 1 and Table 2 detail device use across patient populations and clinical services.

**Table 1:** Technology use across the 12-month period by patient primary health condition.

Device category (Number of devices)		Patient primary health condition								Total	
		Stroke	SCI^a^	Brain injury	Other acquired neurological	Progressive neurological	Congenital neurological	Other			
**Lower limb, n (%)^b^**											
	BWS^c^-T^d^-VR^e^ (1)	254 (13)	84 (10)	125 (26)	14 (13)	55 (9)	9 (7)	39 (42)	580 (14)		
	BWS-OG^f^ (2)	225 (11)	69 (8)	117 (24)	14 (13)	95 (16)	23 (19)	12 (13)	555 (13)		
	Robotic-OG (1)	239 (12)	151 (18)	45 (9)	7 (7)	90 (15)	5 (4)	0 (0)	537 (13)		
	Sensors-VR (2)	119 (6)	64 (8)	31 (6)	16 (15)	68 (11)	12 (10)	14 (15)	324 (8)		
	Robotic-T-BWS-VR (1)	106 (5)	71 (9)	19 (4)	3 (3)	61 (10)	4 (3)	0 (0)	264 (6)		
	Robotic-FES^g^ (1)	40 (2)	127 (15)	19 (4)	1 (1)	63 (10)	3 (3)	0 (0)	253 (6)		
	Total lower limb (8)	—^h^	—	—	—	—	—	—	2513 (60)		
**Upper limb, n (%)^h^**											
	Robotic-VR (4)	562 (28)	106 (13)	32 (7)	6 (6)	53 (9)	26 (21)	1(1)	786 (19)		
	Sensors-VR (4)	251 (13)	77 (9)	23 (5)	28 (27)	53 (9)	32 (26)	20 (22)	484 (12)		
	Total upper limb (8)	—	—	—	—	—	—	—	1270 (30)		
**Other, n (%)^h^**											
	Augmented VR (1)	116 (6)	33 (4)	43 (9)	4 (4)	38 (6)	3 (2)	2 (2)	239 (6)		
	Advanced FES (2)	47 (2)	39 (5)	33 (7)	2 (2)	16 (3)	4 (3)	4 (4)	145 (3)		
	Immersive VR (1)	14 (1)	5 (1)	0 (0)	10 (10)	12 (2)	0 (0)	0 (0)	41 (1)		
	Total other (4)	—	—	—	—	—	—	—	425 (10)		
Total, n		1973	826	487	105	604	121	92	4208		

^a^SCI: spinal cord injury.

^b^Percentages are based on column counts.

^c^BWS: body weight support.

^d^T: treadmill.

^e^VR: virtual reality.

^f^OG: overground.

^g^FES: functional electrical stimulation.

^h^Not applicable.

**Table 2:** Technology use across the 12-month period by clinical service.

Device category (number of devices)		Clinical service			Total	
		Inpatient (onsite)	Outpatient (on-site)	Community (offsite)		
**Lower limb, n (%)^a^**						
	BWS^b^-T^v^-VR^d^ (1)	13 (5)	567 (14)	0 (0)	580 (14)	
	BWS-OG^e^ (2)	74 (28)	481 (12)	0 (0)	555 (13)	
	Robotic-OG (1)	50 (19)	487 (12)	0 (0)	537 (13)	
	Sensors-VR (2)	0 (0)	324 (8)	0 (0)	324 (8)	
	Robotic-T-BWS-VR (1)	9 (3)	255 (6)	0 (0)	264 (6)	
	Robotic-FES^f^ (1)	32 (12)	221 (6)	0 (0)	253 (6)	
	Total lower limb (8)	—^g^	—	—	2513 (60)	
**Upper limb, n (%)^a^**						
	Robotic-VR (4)	47 (18)	739 (19)	0 (0)	786 (19)	
	Sensors-VR (4)	8 (3)	472 (12)	4 (100)	484 (12)	
	Total upper limb (8)	—	—	—	1270 (30)	
**Other, n (%)^a^**						
	Augmented VR (1)	17 (6)	222 (6)	0 (0)	239 (6)	
	Advanced FES (2)	13 (5)	132 (3)	0 (0)	145 (3)	
	Immersive VR (1)	1 (0)	40 (1)	0 (0)	41 (1)	
	Total other (4)	—	—	—	425 (10)	
Total		264	3940	4	4208	

^a^Percentages are based on column counts.

^b^BWS: body weight support.

^c^T: treadmill.

^d^VR: virtual reality.

^e^OG: overground.

^f^FES: functional electrical stimulation.

^g^Not applicable.

### Who Used the Advanced Technology?

#### Clinicians

**Figure 2:**
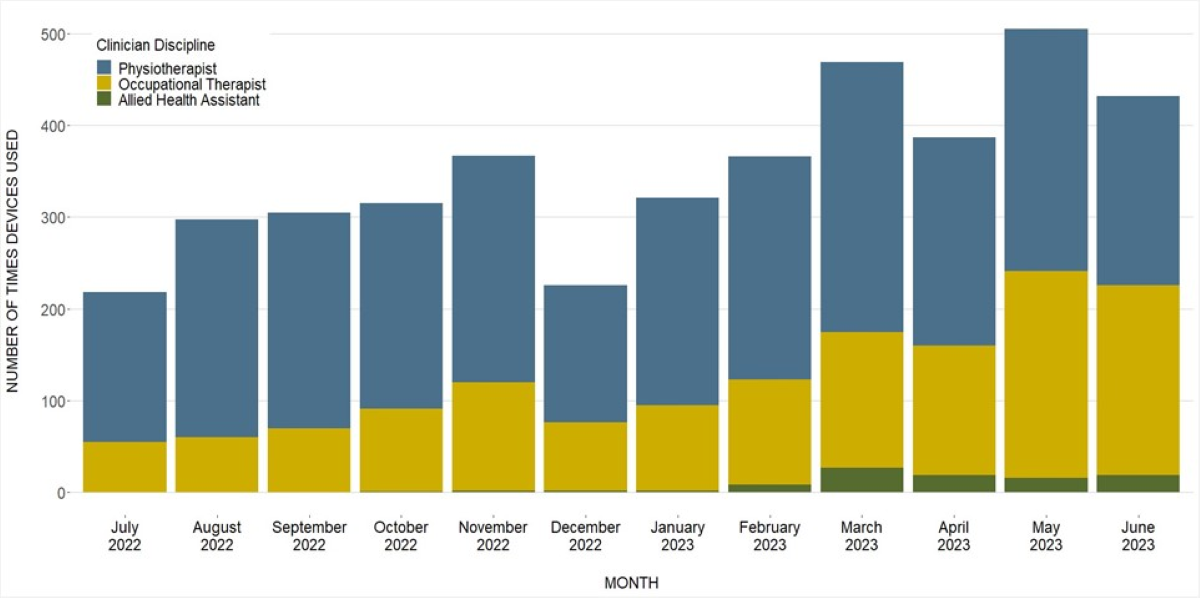
Monthly device use across the 12-month period (July 2022 to June 2023) by clinician discipline, demonstrating an increasing proportion of device use by occupational therapists.

Physiotherapists were the highest users of advanced technology (2716/4208, 65%), followed by occupational therapists (1396/4208, 33%), and allied health assistants (96/4208, 2%). Occupational therapists gradually increased device use each month, relative to physiotherapists (from 25% in the first month to 48% by the twelfth month, Figure 2).

#### Patients

Over 12 months, 445 inpatients and 1502 outpatient and community patients received rehabilitation services. Out of the 269 patients with whom clinicians used advanced technology, the average age of patients was 52.3 years (SD 14.1, range 14-83 years), 95 were female (35%) and 255 had neurological diagnoses (95%); predominantly stroke, spinal cord injury, or brain injury (71%). Table 3 displays patient demographics.

The majority of patients (178/269, 66%) used more than 1 device, including 20% of whom used 5 or more devices. Approximately 45% of patients had 5 or less sessions with advanced technology (120/269), 23% had 6 to 10 sessions (62/269) and 32% had more than 10 sessions (87/269). Data on repeated device use (ie, the same patient using a given device more than once) demonstrated a median of 5 (IQR 1-7) repeated uses overall across devices. The Robotic-T-BWS-VR device had the highest repeated uses (median 7, IQR 5-10), where 89% (32/36) of patients who used this device did so more than once. This was followed closely by the Robotic-OG device (median 7, IQR 4-12), where 81% (52/64) of patients who used this device did so more than once. Details regarding repeated use across each device are summarized in Multimedia Appendix 3.

**Figure 3:**
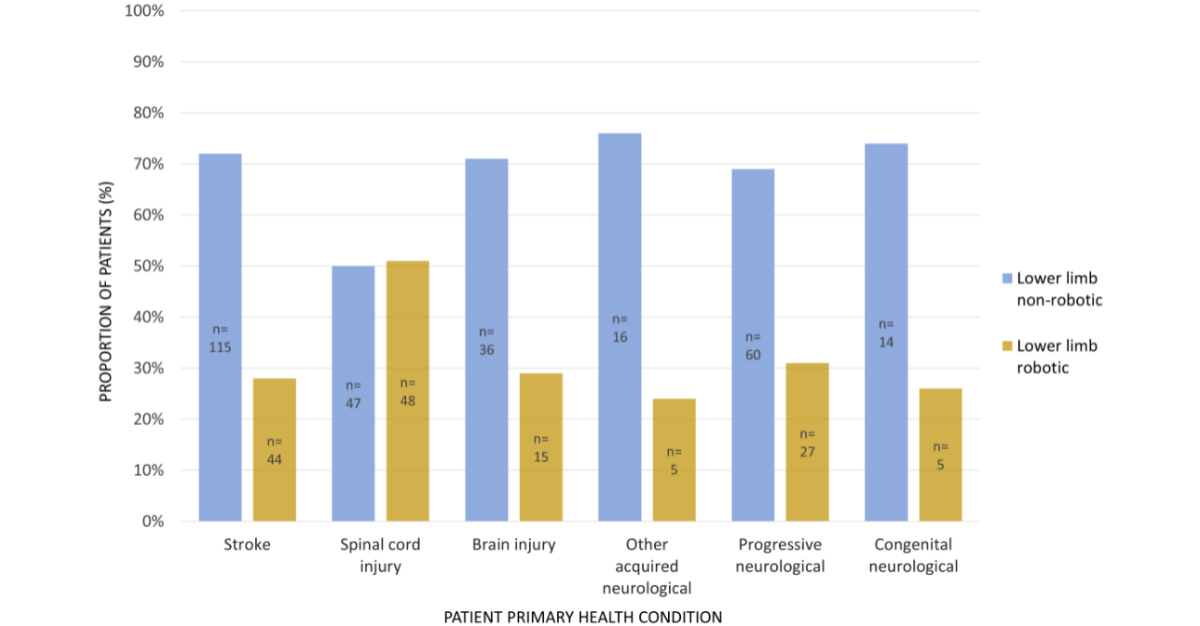
Proportion of patients using robotic versus nonrobotic lower limb devices, grouped by patient primary health condition.

There is strong evidence that use of robotic versus nonrobotic lower limb devices is associated with patient primary health condition (χ^2^_5_=16.8, *P*=.005). For patients with spinal cord injury, clinicians used robotic and nonrobotic lower limb devices equally. For all other conditions, clinicians typically used nonrobotic lower limb devices (between 69% and 76%). Figure 3 depicts these differences.

For upper limb devices, there is no evidence of an association between type of device used (robotic vs nonrobotic) and primary health condition (χ^2^_4_=1.1, *P*=.78). Almost all patients with nonneurological conditions only used sensor and BWS devices, with only 1 having used a robotic upper limb device.

**Table 3:** Demographics of patients who clinicians used advanced technology with (n=269).

Patient demographics				Values
Patient age (years) mean (SD), range			52.3 (14.1), 14-83	
**Patient sex, n (%)**				
	Male	174 (65)		
	Female	95 (35)		
**Patient primary health condition, n (%)**				
	Stroke	88 (33)		
	Spinal cord injury	66 (25)		
	Brain injury	34 (13)		
	Multiple sclerosis	15 (6)		
	Neuro-oncology	9 (3)		
	Cerebral palsy	8 (3)		
	Musculoskeletal	8 (3)		
	Parkinson disease	7 (3)		
	Muscular dystrophy	6 (2)		
	Other neurological conditions	22 (8)		
	Other	6 (2)		
**Patient primary health condition by group^a^; n (%)**				
	Acquired	216 (80)		
	Progressive	42 (16)		
	Congenital	11 (4)		

^a^Acquired conditions include stroke, spinal cord injury, brain injury, musculoskeletal, neuro-oncology, functional neurological disorder, amputee, polio, autoimmune disease, spinal cord injury and brain injury (combined), acute transverse myelitis, deconditioning, cauda equina, and burns. Progressive conditions include multiple sclerosis, Parkinson disease, muscular dystrophy, Multiple systems atrophy, ataxia, spinal muscular atrophy, autosomal dominant adrenoleukodystophy, motor neurone disease, Charcot Marie tooth disease, Parkinson syndrome, neuromyelitis optica, and myositis. Congenital conditions include cerebral palsy, spinal bifida, and heredity spastic paraplegia.

#### Rehabilitation Service Type

Outpatient rehabilitation services were dominant users of advanced technology (3936/4208, 94%). Inpatient services used advanced technology 264 times (6%), while offsite community services used advanced technology 4 times (0.1%). Inpatient clinicians used advanced technology with 49 of 445 patients (11%), while onsite outpatient and community clinicians used advanced technology with 226 of 1502 patients (15%).

**Figure 4:**
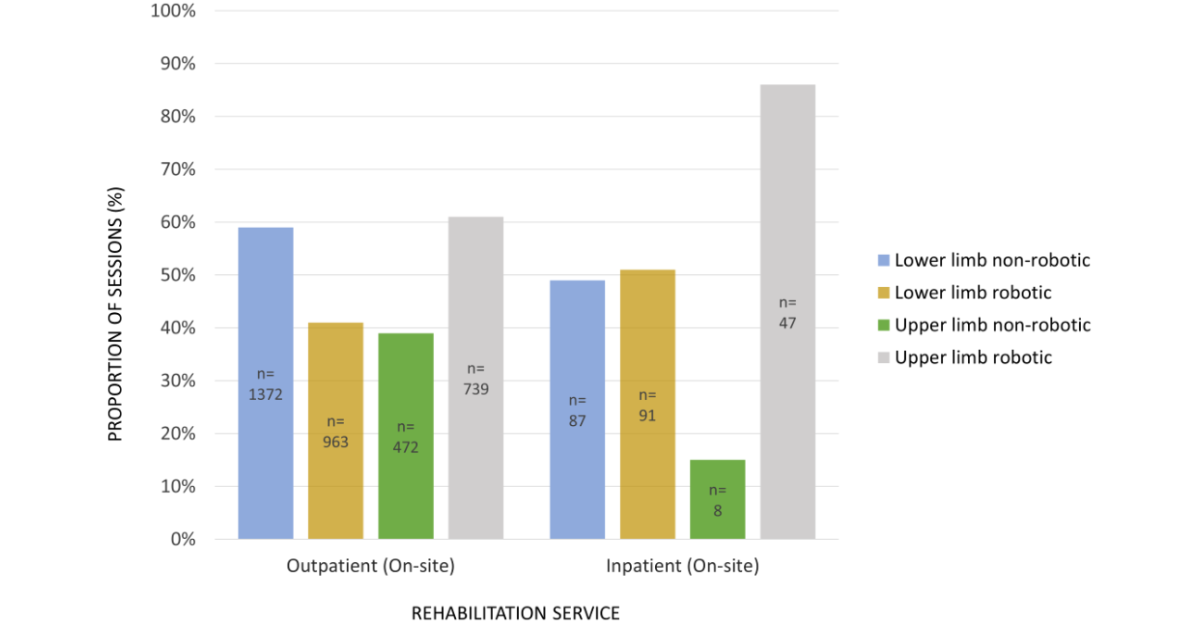
Proportion of sessions in which robotic versus nonrobotic lower and upper limb devices were used, grouped by rehabilitation service.

There is strong evidence of an association between type of lower limb device used (robotic vs nonrobotic) and rehabilitation service (χ^2^_1_=6.6, *P*=.01). Outpatient clinicians used more nonrobotic lower limb devices (1372/2335, 59%), whereas inpatient clinicians used robotic (91/178, 51%) and nonrobotic (87/178, 49%) lower limb devices equally. There is also very strong evidence of an association between type of upper limb device used (robotic vs nonrobotic) and rehabilitation service (χ^2^_1_=13.3, *P*<.001). Inpatient clinicians used robotic upper limb devices (47/55, 85%) considerably more than nonrobotic upper limb devices (8/55, 15%). Meanwhile, outpatient clinicians also used robotic upper limb devices (739/1211, 61%) more than nonrobotic upper limb devices (472/1211, 39%), however the difference was not as prominent. Figure 4 portrays these differences. The relative use of augmented or immersive VR and advanced FES devices was similar across all onsite services. Offsite community services only used portable upper limb sensor devices (4/4, 100%).

### Why Did Clinicians Use Advanced Technology?

Clinicians reported 144 different goals for using advanced technology in patient therapy sessions. On average, clinicians reported 1.8 goals per session (SD 1.2, range 1-5), with only 41 sessions (41/4208, 1%) missing goal documentation. Goals were predominantly at the impairment (5063/8499, 60%) or activity-limitation level (2600/8499, 31%). Although more participation-level goals were reported as the year progressed, these only formed 1% of goals across the 12 months (most commonly return to employment [16/8499, 0.2%]). Clinicians frequently cited goals related to gait training (1959/8499, 23%), lower limb strengthening (555/8499, 7%) and balance (537/8499, 6%). Upper limb goals, particularly fine motor control (942/8499, 11%), range of motion (827/8499, 10%), and strengthening (690/8499, 8%) were also frequently reported. Table 4 details advanced technology therapy session goals.

**Table 4:** Advanced technology therapy session goals.

Type of goal		Sessions, n (%)	ICF^a^ categories (sessions), n (%)			
			Impairment	Activity limitations	Participation restriction	Other
**Lower limb**		3821 (45)	2581 (30)	987 (12)	—^b^	253 (3)
	Gait training	1959 (23)	1211 (14)	498 (6)	—	250 (3)
	Strengthening	555 (7)	555 (7)	—	—	—
	Balance	537 (6)	537 (6)	—	—	—
	Lower limb–specific activities^c^	489 (6)	—	489 (6)	—	—
	Range of motion	127 (1)	127 (1)	—	—	—
	Spasticity	92 (1)	92 (1)	—	—	—
	Coordination	44 (1)	44 (1)	—	—	—
	Sensory	13 (0.15)	13 (0.15)	—	—	—
	Other	5 (0.06)	2 (0.02)	—	—	3 (0.04)
**Upper limb**		3092 (36)	2000 (24)	853 (10)	—	239 (3)
	Fine motor control	942 (11)	283 (3)	659 (8)	—	—
	Range of motion	827 (10)	824 (10)	3 (0.04)	—	—
	Strengthening	690 (8)	690 (8)	—	—	—
	Spasticity	150 (2)	150 (2)	—	—	—
	Gross motor control	97 (1)	—	97 (1)	—	—
	Upper limb–specific activities^d^	94 (1)	—	94 (1)	—	—
	Sensory	52 (0.6)	52 (0.6)	—	—	—
	Unspecified or other	240 (3)	1 (0.01)	—	—	239 (3)
**ADLs^e^ and participation**		650 (8)	—	598 (7)	52 (1)	—
	Personal ADL	497 (6)	—	497 (6)	—	—
	Domestic ADL	71 (1)	—	71 (1)	—	—
	Occupation-related	23 (0.3)	—	—	23 (0.3)	—
	Recreation-related	21 (0.2)	—	—	21 (0.2)	—
	Other	38 (0.5)	—	30 (0.4)	8 (0.09)	—
**Other impairments and activities**		519 (6)	410 (5)	90 (1)	—	19 (0.2)
	Fitness	148 (2)	148 (2)	—	—	—
	Cognition	119 (1)	113 (1)	6 (0.1)	—	—
	Trunk strength or control	86 (1)	60 (0.7)	26 (0.3)	—	—
	Psychological	37 (0.5)	13 (0.2)	24 (0.3)	—	—
	Transfers	33 (0.4)	—	33 (0.4)	—	—
	Bone density	30 (0.4)	30 (0.4)	—	—	—
	Pain	15 (0.2)	15 (0.2)	—	—	—
	Visual scanning	15 (0.2)	15 (0.2)	—	—	—
	Other	36 (0.4)	16 (0.2)	1 (0.01)	—	19 (0.2)
Trial device or equipment		273 (3)	—	—	—	273 (3)
Clinical assessment		144 (2)	72 (1)	72 (1)	—	—^b^
Total^f^		8499 (100)	5063 (60)	2600 (31)	52 (1)	784 (9)

^a^ICF: International Classification of Functional, Disability and Health framework.

^b^Not applicable.

^c^Lower limb–specific activities*:* sit-to-stand training, standing tolerance, running training, stair training, high level mobility (jumping, hopping, skipping), dual tasking

^d^Upper limb–specific activities*:* typing, handwriting, using the computer, using the computer mouse, texting, phone use.

^e^ADL: activities of daily living.

^f^Total number of observations (n) is large as clinicians reported more than one goal for most therapy sessions. Percentages may not add up to 100 due to rounding.

### What Therapy Dosage Did Patients Achieve With Advanced Technology?

Therapy dosage data were collected from progress note entries, in which clinicians reported data from devices. Therapy dosage data from each device are summarized in Multimedia Appendix 4, displaying median active therapy time for 16 devices (80%), median total time on device for 18 devices (90%) and median number of repetitions for 5 devices (25%). However, due to inconsistent device measuring and reporting of therapy dosage data, there were large amounts of missing data and results should be interpreted with caution.

Overall, median total time on devices was 30 minutes (IQR 28-45 minutes), while median device active therapy time was 20 minutes (IQR 13-28 minutes). Active time for lower limb devices was highest for the robotic treadmill BWS device with VR (median 30 minutes, IQR 23-37 minutes), followed by the robotic device with integrated FES (median 27 minutes, IQR 21-33 minutes), and the VR treadmill with optional BWS (median 24 minutes, IQR 16-32 minutes). Drawing conclusions regarding active therapy time relative to total therapy time for lower limb devices was particularly challenging due to inadequate recording of either total time or active time. An OG robotic exoskeleton was the only lower limb device which consistently recorded both active therapy time and total therapy time. This showed approximately 50% of the time spent on the device was active, where median active time achieved in a session was 17 minutes (IQR 13-22 minutes) and total time on the device was 35 minutes (IQR 28-40 minutes).

Active time for upper limb devices was highest for a robotic device with VR used to train the proximal upper limb (median 24 minutes, IQR 16-32 minutes), followed by a sensor-based VR upper limb device (median 20 minutes, IQR 17-20 minutes). There were no marked differences in median active therapy time between robotic and sensor-based upper limb devices. Generally, active therapy time relative to total time on the device was roughly similar across upper limb devices, from between 53% and 67% of time spent active.

A more accurate measure of therapy dosage is number of repetitions or steps, and meters walked [38]. However, this was only reported by four lower limb devices and 1 advanced FES device. No upper limb devices consistently reported number of repetitions. Across the 4 lower limb devices, the range of median repetitions was 356 to 1292 repetitions per session. The highest repetitions and meters walked per session were achieved through the robotic treadmill BWS device with VR (median 1292 repetitions, IQR 994-1674 repetitions; median 756 meters, IQR 573-983 meters).

## Discussion

### Principal Results

This longitudinal observational study investigated the implementation of advanced technologies in a clinical rehabilitation setting. A total of 20 devices were used by clinicians 4208 times, with 269 patients in the first year of the center opening. Frequently used devices included gait training BWS (VR treadmill and OG), OG robotic exoskeletons, and upper limb robotic VR devices. Device use differed considerably between rehabilitation settings, with the majority (3940/4208, 94%) from outpatient services. Clinicians used advanced technologies with a wide range of patients, aged 14 to 83 years, the majority diagnosed with neurological conditions (255/269, 95%). Devices were most often used to target impairment and activity limitation–related goals. Conclusions regarding therapy dosage were limited due to inconsistent recording across devices. These insights derived within a rehabilitation setting can directly inform clinical practice and future research in the field.

Our study demonstrates that while clinicians can successfully incorporate advanced technology into practice, this differs between inpatient, outpatient, and community settings. Notably, inpatient services only accounted for 15% of device use and offsite community services accounted for less than 1%. Reasons for this are likely multifactorial, including marked differences in the available number of inpatients (n=445) compared with outpatient and community patients (n=1502). Coupled with the need to address other goals such as equipment prescription and discharge planning, inpatient clinicians used advanced technology less frequently. Community therapists based offsite showed little use of advanced technologies despite availability of portable devices (n=8). Logistical challenges to accessing and planning device use, and differing service models (eg, focus on hospital-to-home transitions) are likely reasons. These factors collectively influenced inpatient and community-based clinicians’ opportunities to use advanced technology and familiarity with devices. Organizational factors (eg, clinician training and support personnel) also likely contributed to variations between settings, which are being investigated in a separate qualitative study.

Advanced technology use also differed across patient populations and rehabilitation settings. Clinicians most commonly used advanced technologies for patients with stroke (88/269, 47%), and the greatest variety of devices (n=20) for patients with stroke and spinal cord injury. The relative use of lower limb robotic devices was greater for patients with spinal cord injury. Meanwhile, the relative use of both upper limb and lower limb robotic devices was greater in inpatient services. One possible explanation is the nature of impairments in spinal cord injury and acute or subacute neurological conditions in inpatient settings. Clinicians often seek to promote neuroplasticity and address impairments such as muscle weakness through high repetitions, which robotic devices can facilitate [29,30]. Furthermore, the manual handling benefits and opportunities for therapy with robotic devices are potentially greater in these settings and populations, where muscle weakness can be significant [22,39,40]. Although a key argument for using technology in rehabilitation is to increase therapy dosage [6,14,15], our study has highlighted that in practice, advanced technology prescription is highly variable and measuring actual therapy dosage is challenging. Digital health developers should address this as a priority through designing devices which report and standardize dosage output. Devices should include measures regarding the number of repetitions and time spent active, and ensure this information is visible and easily accessible to clinicians. This has important implications for facilitating rigorous research to guide rehabilitation technology prescription and recommended dosage. Overall, further research is required to provide guidance on device selection and prescription across rehabilitation settings and populations. Importantly, future research should seek to establish the effectiveness of using advanced technology alongside conventional interventions to improve patient outcomes in rehabilitation.

Despite the range of devices in the study site, not all devices were used equally. While devices targeting gait training and upper limb impairments were used frequently, advanced functional electrical stimulation and immersive VR devices were used least. In addition, 1 upper limb sensor-VR rehabilitation device was not used at all by clinicians. Reasons for this are likely related to clinician preference and device functionality, both of which warrant investigation in future research. Another possible explanation is the level and reach of clinician training on these devices, which was lower relative to other devices. There was no formal training process for using the upper limb sensor-VR device, and immersive VR and advanced FES were 2 of 5 devices with no increase in number of clinicians trained between baseline and 12 months (n=13 and 11, respectively). With a busy clinical workload and many devices to learn, there are fundamental complexities to providing clinician training within clinical practice which merits further research. Interestingly, there was no evidence in our study of clinicians preferring devices with easier or quicker training processes, reflected in the fact that among the highest used devices was the robotic OG exoskeleton which had the longest training requirements (30-35 hours). A scoping review conducted by our team found training and educating clinicians were the most common strategies used to implement digital interventions in rehabilitation [27]. With evidence to show this is an important element for technology implementation, future research should focus on how training should be conducted, the impact of training on clinician uptake of technology, and determining models of training that are efficient, cost-effective, and sustainable.

### Comparison With Previous Work

Other studies have investigated clinical use of advanced technologies, mostly focusing on OG robotic exoskeleton devices [39,41,42]. These studies also found competing priorities in inpatient settings, where discharge planning often took precedence. In contrast to our study, Gillespie et al [41] found patients with brain injury used the robotic exoskeleton more than patients with stroke or spinal cord injury. However, it was also reported that patients with brain injury had the most variability in therapy dosage achieved with the robotic device [41]. Swank et al [42] also highlighted differences in OG robotic exoskeleton use between patients with spinal cord injury and stroke. Our study extends these findings across a broad spectrum of advanced technologies and neurological conditions. While our study found advanced technologies were primarily used to address impairments and activity limitations, Putrino and Krakauer [43] argue that neurological rehabilitation technologies are best used to address impairments, and conventional rehabilitation is better at targeting activity and participation-level goals. Therefore, it is important that use of technology is combined with conventional rehabilitation methods, rather than using technology in isolation [6,20,22]. Finally, our study found clinicians using advanced technologies with only 11% of inpatients and 15% of outpatient and community patients. This suggests that current advanced technologies may not be suitable for everyone and is consistent with other literature in the field demonstrating 15%-20% patient eligibility for studies evaluating rehabilitation technologies [5,6]. This emphasizes the need for a better understanding of who technology is appropriate and effective for, and how to design technologies to better meet the needs of both patients and clinicians.

### Limitations

This study has a few limitations. First, a limitation was the inability to capture patient outcome measure data due to a lack of standardized recording and reporting of outcome measures clinically. As such, this study focuses on clinician use and uptake of advanced technology in a real-world rehabilitation setting, rather than the effectiveness of using advanced technology to improve patient outcomes. Inconsistent measuring and recording of outcome measures is a common issue in rehabilitation and should be prioritized for clinical practice and practice-embedded research [44-47]. Second, a multisite study was not feasible given the novelty of the technology center. We sought to address generalizability by collecting data on a wide range of patients, clinical disciplines, and rehabilitation services. However, the authors of this study acknowledge that the study site is a well-resourced, urban, private-public rehabilitation setting within a high-income country. Noteworthy factors which likely contributed to substantial device uptake are the construction of a purpose-built technology therapy center within the existing site, availability of a wide variety of advanced technologies, appointment of an advanced technology lead to support multiple aspects of implementation including staff training, and patient access to long-term rehabilitation services through public funding models. Therefore, lower resourced settings would likely face challenges in applying the findings of this research. Current evidence has shown low-cost devices such as commercially available VR, activity trackers and mobile phone apps are cost-effective in rehabilitation [48]. Future research should investigate the cost-effectiveness of high-cost advanced technologies. Research in the field should also continue to place equal emphasis on affordable and low-cost rehabilitation technologies. Third, data in this study were reliant on clinicians using note templates for documentation. As such, device use is likely underreported, particularly for disciplines such as speech pathology or recreational therapy who were not captured in our data. However, given the large number of advanced technology sessions captured, the data likely adequately reflect clinical practice for physiotherapists and occupational therapists in the setting, currently the main users of advanced technologies.

### Conclusions

This is one of the largest longitudinal observational studies conducted within a real-world rehabilitation setting which successfully implemented numerous advanced robotic and virtual reality technologies for patients with a wide range of health conditions. This study provides valuable insights into the types of advanced technologies most likely to be used in rehabilitation, by which clinical disciplines and rehabilitation service types, for which kind of patients, and for what purpose. Findings are important to inform rehabilitation technology implementation, aiding clinicians in device selection, outlining the likely uptake of devices, and providing recommendations for future research in the field.

## Appendix

Multimedia Appendix 1 Description of devices in the advanced technology therapy center.

Multimedia Appendix 2 Advanced technology therapy session note template (lower limb device example).

Multimedia Appendix 3 Average repeated use per device, by number of sessions and by number of patients (ie. the same patient using a given device more than once).

Multimedia Appendix 4 Summary of therapy dosage data collected from progress note entries, in which clinicians documented data reported on devices.

## Abbreviations

BWS: body-weight support
BWS-T: body-weight support treadmill
FES: functional electrical stimulation
ICF: International Classification of Functional, Disability and Health
OG: overground
RECORD: Reporting of Studies Conducted using Observational Routinely-collected health Data
VR: virtual reality

## Data Availability

The data sets generated and analyzed during this study are not publicly available due to privacy reasons. Deidentified data sets are available from the corresponding author on reasonable request and with approval from the ethics committee and study site.

## References

[1] (2019). WHO guideline: recommendations on digital interventions for health system strengthening. World Health Organization.

[2] (2019). Rehabilitation 2030 initiative. World Health Organization.

[3] (2017). The need to scale up rehabilitation. World Health Organization.

[4] Hassett L, Allen N, van den Berg M, Hayre CM, Muller D, Scherer M (2020). Feedback-based technologies for adult physical rehabilitation. Everyday Technologies in Healthcare.

[5] Hassett L, van den Berg M, Lindley RI, Crotty M, McCluskey A, van der Ploeg HP, Smith ST, Schurr K, Howard K, Hackett ML, Killington M, Bongers B, Togher L, Treacy D, Dorsch S, Wong S, Scrivener K, Chagpar S, Weber H, Pinheiro M, Heritier S, Sherrington C (2020). Digitally enabled aged care and neurological rehabilitation to enhance outcomes with Activity and MObility UsiNg Technology (AMOUNT) in Australia: A randomised controlled trial. PLoS Med.

[6] Laver KE, Lange B, George S, Deutsch JE, Saposnik G, Crotty M (2017). Virtual reality for stroke rehabilitation. Cochrane Database Syst Rev.

[7] Leow XRG, Ng SLA, Lau Y (2023). Overground robotic exoskeleton training for patients with stroke on walking-related outcomes: a systematic review and meta-analysis of randomized controlled trials. Arch Phys Med Rehabil.

[8] Yang X, Shi X, Xue X, Deng Z (2023). Efficacy of robot-assisted training on rehabilitation of upper limb function in patients with stroke: a systematic review and meta-analysis. Arch Phys Med Rehabil.

[9] Mirelman A, Rochester L, Maidan I, Del Din S, Alcock L, Nieuwhof F, Rikkert MO, Bloem BR, Pelosin E, Avanzino L, Abbruzzese G, Dockx K, Bekkers E, Giladi N, Nieuwboer A, Hausdorff JM (2016). Addition of a non-immersive virtual reality component to treadmill training to reduce fall risk in older adults (V-TIME): a randomised controlled trial. Lancet.

[10] Hamilton C, McCluskey A, Hassett L, Killington M, Lovarini M (2018). Patient and therapist experiences of using affordable feedback-based technology in rehabilitation: a qualitative study nested in a randomized controlled trial. Clin Rehabil.

[11] Barry G, Galna B, Rochester L (2014). The role of exergaming in Parkinson's disease rehabilitation: a systematic review of the evidence. J Neuroeng Rehabil.

[12] Vaughan-Graham J, Brooks D, Rose L, Nejat G, Pons J, Patterson K (2020). Exoskeleton use in post-stroke gait rehabilitation: a qualitative study of the perspectives of persons post-stroke and physiotherapists. J Neuroeng Rehabil.

[13] Klaic M, Fong J, Crocher V, Davies K, Brock K, Sutton E, Oetomo D, Tan Y, Galea MP (2024). Application of the extended technology acceptance model to explore clinician likelihood to use robotics in rehabilitation. Disabil Rehabil Assist Technol.

[14] Allen NE, Song J, Paul SS, Smith S, O'Duffy J, Schmidt M, Love R, Sherrington C, Canning CG (2017). An interactive videogame for arm and hand exercise in people with Parkinson's disease: a randomized controlled trial. Parkinsonism Relat Disord.

[15] Zhang B, Wong KP, Kang R, Fu S, Qin J, Xiao Q (2023). Efficacy of robot-assisted and virtual reality interventions on balance, gait, and daily function in patients with stroke: a systematic review and network meta-analysis. Arch Phys Med Rehabil.

[16] Cresswell K, Cunningham-Burley S, Sheikh A (2018). Health care robotics: qualitative exploration of key challenges and future directions. J Med Internet Res.

[17] Gomes M, Murray E, Raftery J (2022). Economic evaluation of digital health interventions: methodological issues and recommendations for practice. Pharmacoeconomics.

[18] Morone G, Cocchi I, Paolucci S, Iosa M (2020). Robot-assisted therapy for arm recovery for stroke patients: state of the art and clinical implication. Expert Rev Med Devices.

[19] Pilli K, Worne B, Simpson G (2023). Clinician experiences with using assistive technology in brain injury rehabilitation: a survey of clinician capability, attitudes, and barriers. Brain Impair.

[20] Langan J, Subryan H, Nwogu I, Cavuoto L (2018). Reported use of technology in stroke rehabilitation by physical and occupational therapists. Disabil Rehabil Assist Technol.

[21] Glegg SMN, Levac DE (2018). Barriers, facilitators and interventions to support virtual reality implementation in rehabilitation: a scoping review. PM R.

[22] Lo K, Stephenson M, Lockwood C (2020). Adoption of robotic stroke rehabilitation into clinical settings: a qualitative descriptive analysis. JBI Evid Implement.

[23] Mitchell J, Shirota C, Clanchy K (2023). Factors that influence the adoption of rehabilitation technologies: a multi-disciplinary qualitative exploration. J Neuroeng Rehabil.

[24] Greenhalgh T, Wherton J, Papoutsi C, Lynch J, Hughes G, A'Court C, Hinder S, Fahy N, Procter R, Shaw S (2017). Beyond adoption: a new framework for theorizing and evaluating nonadoption, abandonment, and challenges to the scale-up, spread, and sustainability of health and care technologies. J Med Internet Res.

[25] Gallagher R, Zhang L (2021). Evaluating mobile health technologies: does the traditional randomized controlled trial serve our needs?. Eur J Cardiovasc Nurs.

[26] Charette C, Déry J, Blanchette AK, Faure C, Routhier F, Bouyer LJ, Lamontagne M (2023). A systematic review of the determinants of implementation of a locomotor training program using a powered exoskeleton for individuals with a spinal cord injury. Clin Rehabil.

[27] Pearce L, Costa N, Sherrington C, Hassett L (2023). Implementation of digital health interventions in rehabilitation: a scoping review. Clin Rehabil.

[28] Jarvis K, Thetford C, Turck E, Ogley K, Stockley RC (2024). Understanding the barriers and facilitators of digital health technology (DHT) implementation in neurological rehabilitation: an integrative systematic review. Health Serv Insights.

[29] Gassert R, Dietz V (2018). Rehabilitation robots for the treatment of sensorimotor deficits: a neurophysiological perspective. J Neuroeng Rehabil.

[30] Molteni F, Gasperini G, Cannaviello G, Guanziroli E (2018). Exoskeleton and end-effector robots for upper and lower limbs rehabilitation: narrative review. PM R.

[31] Lohse KR, Hilderman CGE, Cheung KL, Tatla S, Van der Loos HFM (2014). Virtual reality therapy for adults post-stroke: a systematic review and meta-analysis exploring virtual environments and commercial games in therapy. PLoS One.

[32] Miller LE, Zimmermann AK, Herbert WG (2016). Clinical effectiveness and safety of powered exoskeleton-assisted walking in patients with spinal cord injury: systematic review with meta-analysis. Med Devices (Auckl).

[33] Turchetti G, Vitiello N, Trieste L, Romiti S, Geisler E, Micera S (2014). Why effectiveness of robot-mediated neurorehabilitation does not necessarily influence its adoption. IEEE Rev Biomed Eng.

[34] Horn SD, DeJong G, Deutscher D (2012). Practice-based evidence research in rehabilitation: an alternative to randomized controlled trials and traditional observational studies. Arch Phys Med Rehabil.

[35] Benchimol EI, Smeeth L, Guttmann A, Harron K, Moher D, Petersen I, Sørensen HT, von Elm E, Langan SM, RECORD Working Committee (2015). The REporting of studies conducted using observational routinely-collected health data (RECORD) statement. PLoS Med.

[36] Aprile I, Cruciani A, Germanotta M, Gower V, Pecchioli C, Cattaneo D, Vannetti F, Padua L, Gramatica F (2019). Upper limb robotics in rehabilitation: an approach to select the devices, based on rehabilitation aims, and their evaluation in a feasibility study. Appl Sci.

[37] International classification of functioning, disability and health: ICF. World Health Organization.

[38] Scrivener K, Sherrington C, Schurr K (2012). Exercise dose and mobility outcome in a comprehensive stroke unit: description and prediction from a prospective cohort study. J Rehabil Med.

[39] Heinemann AW, Jayaraman A, Mummidisetty CK, Spraggins J, Pinto D, Charlifue S, Tefertiller C, Taylor HB, Chang S, Stampas A, Furbish CL, Field-Fote EC (2018). Experience of robotic exoskeleton use at four spinal cord injury model systems centers. J Neurol Phys Ther.

[40] Postol N, Barton J, Wakely L, Bivard A, Spratt NJ, Marquez J (2024). "Are we there yet?" expectations and experiences with lower limb robotic exoskeletons: a qualitative evaluation of the therapist perspective. Disabil Rehabil.

[41] Gillespie J, Arnold D, Trammell M, Bennett M, Ochoa C, Driver S, Callender L, Sikka S, Dubiel R, Swank C (2023). Utilization of overground exoskeleton gait training during inpatient rehabilitation: a descriptive analysis. J Neuroeng Rehabil.

[42] Swank C, Trammell M, Bennett M, Ochoa C, Callender L, Sikka S, Driver S (2020). The utilization of an overground robotic exoskeleton for gait training during inpatient rehabilitation-single-center retrospective findings. Int J Rehabil Res.

[43] Putrino D, Krakauer JW (2023). Neurotechnology's prospects for bringing about meaningful reductions in neurological impairment. Neurorehabil Neural Repair.

[44] Pohl J, Held JPO, Verheyden G, Alt Murphy M, Engelter S, Flöel A, Keller T, Kwakkel G, Nef T, Ward N, Luft AR, Veerbeek JM (2020). Consensus-based core set of outcome measures for clinical motor rehabilitation after stroke-a delphi study. Front Neurol.

[45] Tyson S, Watson A, Moss S, Troop H, Dean-Lofthouse G, Jorritsma S, Shannon M, Greater Manchester Outcome Measures Project (2008). Development of a framework for the evidence-based choice of outcome measures in neurological physiotherapy. Disabil Rehabil.

[46] Skinner A, Turner-Stokes L (2006). The use of standardized outcome measures in rehabilitation centres in the UK. Clin Rehabil.

[47] Colquhoun HL, Lamontagne M, Duncan EA, Fiander M, Champagne C, Grimshaw JM (2017). A systematic review of interventions to increase the use of standardized outcome measures by rehabilitation professionals. Clin Rehabil.

[48] Pinheiro MB, Hassett L, Sherrington C, Hayes A, van den Berg M, Lindley RI, Crotty M, Chagpar S, Treacy D, Weber H, Fairhall N, Wong S, McCluskey A, Togher L, Scrivener K, Howard K (2023). Economic evaluation of digitally enabled aged and neurological rehabilitation care in the activity and mobility using technology (AMOUNT) trial. Clin Rehabil.

