# Supplementary material for: Advanced Technology in a Real-World Rehabilitation Setting: Longitudinal Observational Study on Clinician Adoption and Implementation
Source: J Med Internet Res. 2024 Dec 30;26:e60374. doi: 10.2196/60374 (PMC11729780; doi:10.2196/60374)
Supplement: Multimedia Appendix 1 [file jmir_v26i1e60374_app1.docx]

**Appendix 1.** Description of devices within the advanced technology therapy centre

| **Type of device** | **Device category** | **Device/s** | **Company** | **Device description** | **Clinician training requirement**  (NB: numbered cells = multi-step process to training, bullet points = different training model options) |
| --- | --- | --- | --- | --- | --- |
| **Lower Limb** | **BWS-T-VR** | C-mill VR+ | Hocoma Motek, Amsterdam, The Netherlands | Treadmill with augmented and virtual reality with the option for body-weight support.  <https://www.hocoma.com/solutions/c-mill/> | - External Hocoma trainer: 8 hours - Internal train-the-trainer: 4-5 supervised sessions (5-6 hours) |
|  | **BWS-OG** | Zero G | Aretech, Virginia, United States | Dynamic body-weight support gait and balance system along a ceiling track.  <https://www.aretechllc.com/products/zerog-gait-and-balance/> | - External Aretech trainer: 4 hours - Internal train-the-trainer: 3-4 supervised sessions (4-5 hours) |
|  |  | Andago V2.0 | Hocoma, Volketswil, Switzerland | Overground, robotic dynamic body-weight support system.  <https://www.hocoma.com/solutions/andago/> | - External Hocoma trainer: 4 hours - Internal train-the-trainer: 2-5 supervised sessions (3-6 hours) |
|  | **Robotic-OG** | EksoNR | Ekso Bionics, California, United States | Robotic overground exoskeleton.  <https://eksobionics.com/eksonr/> | - Level 1 certification   (Can operate EksoNR with another certified Ekso physiotherapist)   - - External EksoNR Trainer: 12 hours   - (Internal train-the-trainer combines Level 1 and Level 2 certification, see below) - Level 2 certification   (Can operate EksoNR independently and can train internal physiotherapist for level 1 certification)   - - External EksoNR Trainer: 12 hours   - Internal train-the-trainer: complete detailed training including training checklist and training log, experience with 8+ clients, 30+ supervised sessions, final assessment of a client session in the device (30-35 hours) - Level 3 certification   (As per level 2 certification, and can train and certify therapists to level 2 certification)   - - Must have used EksoNR for 12 months prior to applying for accreditation   - External EksoNR trainer: practical exams, compete 2x level 2 certification and a written 100 question exam (hours undefined) |
|  | **Sensors-VR** | Tymo | Tyromotion, Graz, Austria | Sensor-based portable balance platform.  <https://tyromotion.com/en/products/tymo/> | 1. Online training videos through TyroAcademy^1^ (2-3 hours) 2. Internal train-the-trainer: 2-3 supervised sessions (2-3 hours) |
|  |  | Pablo (Lower extremity) | Tyromotion, Graz, Austria | Sensor-based gait analysis and training system.  <https://tyromotion.com/en/products/pablo-lower-extremity/> | 1. Online training videos through TyroAcademy^1^ (2-3 hours) 2. Internal train-the-trainer: 1-2 supervised sessions (2 hours) |
|  | **Robotic-T-BWS-VR** | Lokomat | Hocoma, Volketswil, Switzerland | Treadmill-based, robotic, body-weight support gait training system.  <https://www.hocoma.com/solutions/lokomat/> | - Basic training   (To qualify as a ‘user’)   - - External Hocoma trainer: 16 hours - Advanced training   (To qualify as a ‘trainer’, 3-6 months after basic training):   - - External Hocoma trainer: 16 hours - Internal train-the-trainer (to qualify as ‘user’): 10-15 sessions (15-25 hours) |
|  | **Robotic-FES** | Erigo Pro | Hocoma, Volketswil, Switzerland | Tilt table with lower limb robotic and eight-channel functional electrical stimulation.  <https://www.hocoma.com/solutions/erigo/> | - External Hocoma trainer: 6 hours - Internal train-the-trainer: 3-5 supervised sessions (3-6 hours) |
| **Upper Limb** | **Robotic-VR** | Amadeo | Tyromotion, Graz, Austria | Robotic finger and hand training system with augmented virtual reality.  <https://tyromotion.com/en/products/amadeo/> | 1. Online training videos through TyroAcademy^1^ (2-3 hours) 2. Train-the-trainer: 3-5 supervised sessions (3-6 hours) |
|  |  | Armeo Power | Hocoma, Volketswil, Switzerland | Robotic upper limb device for gross motor training, with augmented virtual reality. (For severe upper limb impairment)  <https://www.hocoma.com/solutions/armeo-power/> | - Basic training (to qualify as a ‘user’): 8 hours - Advanced training (to qualify as a ‘trainer’, 6 months after basic training): 8 hours |
|  |  | Diego | Tyromotion, Graz, Austria | Robotic upper limb-weight support system with augmented and/or immersive virtual reality.  <https://tyromotion.com/en/products/diego/> | 1. Online training videos through TyroAcademy^1^ (2-3 hours) 2. a) External Tyromotion trainer: 2-4 hours   b) Train-the-trainer: 2-4 hours |
|  |  | H-MAN | Articares, Singapore | Robotic upper limb system with augmented virtual reality.  <https://articares.com/h-man/> | - Internal train-the-trainer: 1-2 supervised sessions (2 hours) |
|  | **Sensors-VR** | Armeo Senso | Hocoma, Volketswil, Switzerland | Sensor-based upper limb device with augmented virtual reality. (For mild upper limb impairment)  <https://www.hocoma.com/solutions/armeo-senso/> | - External Hocoma trainer: 2 hours - Internal train-the-trainer: 3-5 supervised sessions (3-6 hours) |
|  |  | Armeo Spring | Hocoma, Volketswil, Switzerland | Spring-loaded upper limb device (non-powered, passive exoskeleton) for gross motor training, with augmented virtual reality. (For moderate upper limb impairment)  <https://www.hocoma.com/solutions/armeo-spring/> | - External Hocoma trainer: 4 hours - Internal train-the-trainer: 3-5 supervised sessions (3-6 hours) |
|  |  | Pablo (Upper extremity) | Tyromotion, Graz, Austria | Sensor-based upper limb and trunk training system.  <https://tyromotion.com/en/products/pablo/> | 1. Online training videos through TyroAcademy^1^ (2-3 hours) 2. Train-the-trainer: 1-2 supervised sessions (2 hours) |
|  |  | AbleX | AbleX Healthcare, Auckland, New Zealand | Sensor-based, computer-based upper limb device with augmented virtual reality.  <https://ablex.healthcare/public/about> | Internal train-the-trainer: 1-2 supervised sessions (2 hours) |
|  |  | Music glove | FlintRehab, Irvine, California | Instrumented hand glove training system with interactive music-based computer games.  <https://www.flintrehab.com/product/musicglove-hand-therapy/> | (No formal training program)  Informal review of device and practice with a current user (1-2 hours) |
| **Other** | **Augmented VR** | Myro | Tyromotion, Graz, Austria | Interactive, adjustable, large touch screen with a sensor-based surface and augmented virtual reality.  <https://tyromotion.com/en/products/myro/> | 1. Online training videos through TyroAcademy^1^ (2-3 hours) 2. Internal train-the-trainer: 1-2 supervised sessions (2 hours) |
|  | **Advanced FES** | Xcite2 | Restorative Therapies, Maryland, United States | Multi-channel functional electrical stimulation with a wide variety of pre-programmed activities for the upper limb, lower limb and trunk to strengthen muscles and re-train activities of daily living.  <https://restorative-therapies.com/ifes-systems/xcite2/> | 1. Online training videos through Restorative Therapies Academy^2^ (2-3 hours) 2. Internal train-the-trainer: 1-3 supervised sessions (2-3 hours) |
|  |  | RT300 iFES cycle | Restorative Therapies, Maryland, United States | Motorised ergometer cycling machine with multi-channel functional electrical stimulation.  <https://restorative-therapies.com/ifes-systems/rt300/> | 1. Online training videos through Restorative Therapies Academy^2^ (2-3 hours) 2. Internal train-the-trainer: 1-3 supervised sessions (2-3 hours) |
|  | **Immersive VR** | Recovery VR | Recovery VR, Canberra, Australia | Immersive virtual reality headset with interactive hand controls.  <https://recoveryvr.com.au/> | 1. Online training videos (2 hours) 2. Internal train-the-trainer: 1-2 supervised sessions (2 hours) |
| *BWS = Body weight support, T = Treadmill, VR = Virtual reality, OG = Overground, FES = Functional electrical stimulation, LL = Lower limb, UL = Upper Limb* | | | | | |

**References**

1. Tyromotion. TyroAcademy, <https://tyroacademy.tyromotion.com/start> (2024, accessed 10 April 2024).

2. Restorative Therapies. RTILink, <https://www.rtilink.com/datalink/login.htm> (2023, accessed 10 April 2024).
