# Supplementary material for: Advanced Technology in a Real-World Rehabilitation Setting: Longitudinal Observational Study on Clinician Adoption and Implementation
Source: J Med Internet Res. 2024 Dec 30;26:e60374. doi: 10.2196/60374 (PMC11729780; doi:10.2196/60374)
Supplement: Multimedia Appendix 2 [file jmir_v26i1e60374_app2.docx]

**Appendix 2.** Advanced technology therapy session note template (lower limb device example)

| **Subjective**  **Objective**  **Treatment/Therapy**  **Advanced Technology Therapy Session: Lower Limb**  Device(s) used:  Session location:  Session goal(s):  Duration (per device):  Up time/walk time (if applicable):  Step count/meters walked (if applicable):  Games & activities completed:  Adverse event(s):  Skin check:  **Conventional Therapy**  Duration:  Exercise & activities completed:  **Assessment/Recommendations**  **Plan** |
| --- |
