# Supplementary material for: Advanced Technology in a Real-World Rehabilitation Setting: Longitudinal Observational Study on Clinician Adoption and Implementation
Source: J Med Internet Res. 2024 Dec 30;26:e60374. doi: 10.2196/60374 (PMC11729780; doi:10.2196/60374)
Supplement: Multimedia Appendix 3 [file jmir_v26i1e60374_app3.docx]

| Device category | Device | Average number of repeated sessions | | | Average number of patients | |
| --- | --- | --- | --- | --- | --- | --- |
|  |  | Median | IQR | Range | N repeated use/N total | % |
| LL | | | | | | |
| BWS-T-VR | C-mill VR+ | 4 | 7 | 2 – 26 | 78/107 | 73 |
| BWS-OG | Zero G | 4 | 6 | 2 – 27 | 75/102 | 74 |
| BWS-OG | Andago V2.0 | 4 | 3 | 2 – 7 | 6/11 | 55 |
| Robotic-OG | EksoNR | 7 | 8 | 2 – 64 | 52/64 | 81 |
| Sensors-VR | Tymo | 4 | 5 | 2 – 16 | 35/48 | 73 |
| Sensors-VR | Pablo (LL) | 5 | 3 | 2 – 16 | 22/36 | 61 |
| Robotic-T-BWS-VR | Lokomat | 7 | 5 | 2 – 23 | 32/36 | 89 |
| Robotic-FES | Erigo Pro | 6 | 6 | 2 – 24 | 34/44 | 77 |
| UL | | | | | | |
| Robotic-VR | Amadeo | 6 | 7 | 2 – 47 | 39/52 | 75 |
| Robotic-VR | Armeo Power | 6 | 8 | 2 – 46 | 31/40 | 78 |
| Robotic-VR | Diego | 3 | 5 | 2 – 16 | 19/27 | 70 |
| Robotic-VR | H-MAN | 4 | 2 | 2 – 7 | 5/13 | 39 |
| Sensors-VR | Armeo Senso | 3 | 1 | 2 – 10 | 12/18 | 67 |
| Sensors-VR | Armeo Spring | 5 | 4 | 3 – 27 | 11/16 | 69 |
| Sensors-VR | Pablo (UL) | 5 | 7 | 2 – 30 | 40/65 | 62 |
| Sensors-VR | AbleX | 2 | 0 | 2 – 5 | 5/5 | 100 |
| Other | | | | | | |
| Augmented VR | Myro | 4 | 5 | 2 – 23 | 37/60 | 62 |
| Advanced FES | Xcite2 | 4 | 5 | 2 – 15 | 22/40 | 55 |
| Advanced FES | RT300 iFES cycle | 5 | 2 | 3 – 7 | 2/5 | 40 |
| Immersive VR | Recovery VR | 4 | 5 | 2 – 12 | 7/10 | 70 |

**Appendix 3.** Average repeated use per device, by number of sessions and by number of patients (ie. the same patient using a given device more than once)
