# Supplementary material for: Advanced Technology in a Real-World Rehabilitation Setting: Longitudinal Observational Study on Clinician Adoption and Implementation
Source: J Med Internet Res. 2024 Dec 30;26:e60374. doi: 10.2196/60374 (PMC11729780; doi:10.2196/60374)
Supplement: Multimedia Appendix 4 [file jmir_v26i1e60374_app4.docx]

**Appendix 4.** Summary of therapy dosage data collected from progress note entries, in which clinicians documented data reported on devices

| Device category | Device | Therapy dosage per session* | | | | | | | | | | | | |
| --- | --- | --- | --- | --- | --- | --- | --- | --- | --- | --- | --- | --- | --- | --- |
|  |  | Active time on device (minutes) | | | | Total time on device (minutes) | | | | Median active time/ Median total time  (%) | Number of repetitions | | | |
|  |  | Median | IQR | N/ total usage | Missing (%) | Median | IQR | N/ total usage | Missing (%) |  | Median | IQR | N/ total usage | Missing (%) |
| LL | | | | | | | | | | | | | | |
| BWS-T-VR | C-mill VR+ | 24 | 16 | 455/580 | 22 | 30 | 19 | 152/580 | 74 | 80 | 1052 | 850 | 312/580 | 46 |
| BWS-OG | Zero G | N/R | | | | 42 | 20 | 459/526 | 13 | - | N/R | | | |
| BWS-OG | Andago V2.0 | N/R | | | | 28 | 15 | 23/29 | 21 | - | N/R | | | |
| Robotic-OG | EksoNR | 17 | 9 | 530/537 | 1 | 35 | 12 | 535/537 | <1 | 49 | 356 | 192 | 532/537 | 1 |
| Sensors-VR | Tymo | 9 | 9 | 105/197 | 47 | 28 | 10 | 106/197 | 46 | 32 | N/R | | | |
| Sensors-VR | Pablo (LL) | 9 | 8 | 72/127 | 43 | 20 | 15 | 53/127 | 58 | 45 | N/R | | | |
| Robotic-T-BWS-VR | Lokomat | 30 | 14 | 259/264 | 2 | N/R | | | | - | 1292 | 680 | 246/264 | 7 |
| Robotic-FES | Erigo Pro | 27 | 12 | 250/253 | 1 | N/R | | | | - | 500 | 296 | 239/253 | 6 |
| UL | | | | | | | | | | | | | | |
| Robotic-VR | Amadeo | 18 | 8 | 306/374 | 18 | 30 | 10 | 343/374 | 8 | 60 | N/R | | | |
| Robotic-VR | Armeo Power | 24 | 16 | 208/279 | 25 | 45 | 30 | 244/279 | 13 | 53 | N/R | | | |
| Robotic-VR | Diego | 16 | 8 | 83/106 | 22 | 30 | 10 | 88/106 | 17 | 53 | N/R | | | |
| Robotic-VR | H-MAN | 15 | 6 | 22/27 | 19 | 25 | 14 | 13/27 | 48 | 60 | N/R | | | |
| Sensors-VR | Armeo Senso | N/R | | | | 45 | 10 | 43/49 | 12 | - | N/R | | | |
| Sensors-VR | Armeo Spring | 18 | 10 | 73/97 | 25 | 30 | 15 | 88/97 | 9 | 60 | N/R | | | |
| Sensors-VR | Pablo (UL) | 16 | 8 | 209/325 | 36 | 30 | 15 | 277/325 | 15 | 53 | N/R | | | |
| Sensors-VR | AbleX | 20 | 3 | 9/13 | 31 | 30 | 8 | 10/13 | 23 | 67 | N/R | | | |
| Other | | | | | | | | | | | | | | |
| Augmented VR | Myro | 14 | 10 | 117/239 | 51 | 20 | 15 | 201/239 | 16 | 70 | N/R | | | |
| Advanced FES | Xcite2 | N/R | | | | 45 | 20 | 89/132 | 33 | - | 60 | 48 | 75/132 | 43 |
| Advanced FES | RT300 iFES cycle | 23 | 19 | 9/13 | 31 | 52 | 29 | 9/13 | 31 | 44 | N/R | | | |
| Immersive VR | Recovery VR | 20 | 20 | 13/41 | 68 | 40 | 29 | 30/41 | 27 | 50 | N/R | | | |
| Key: LL=Lower limb, UL=Upper limb, BWS=Body weight support, T=Treadmill, VR=Virtual reality, OG=Overground, FES=Functional electrical stimulation,  N/R: Variable not measured or reported by the device, or >75% missing data due to challenges for clinicians accessing data on the device for clinical documentation.  *Four gait training devices also recorded and reported therapy dosage data regarding the number of meters walked within a session on the device:  Zero G: Median: 154 meters, IQR: 188 meters, N/total usage: 466/526, Missing: 11%  C-Mill VR+: Median: 450 meters, IQR: 443 meters, N/total usage: 410/580, Missing: 71%  Lokomat: Median: 756 meters, IQR: 410 meters, N/total usage: 246/264, Missing: 7%,  Andago: Median: 58 meters, IQR: 105 meters, N/total usage: 27/29, Missing: 7% | | | | | | | | | | | | | | |
